# C-854-06. Implementation of a Burn Specific Sepsis Tool

**DOI:** 10.1093/jbcr/irag033.082

**Published:** 2026-04-06

**Authors:** Haven Price, Mario Andrade

**Affiliations:** The University of Kansas Health System Burnett Burn Center, Kansas City, KS; The University of Kansas Health System Burnett Burn Center, Kansas City, KS

## Abstract

**Introduction:**

Due to the hypermetabolic response that occurs in burn injuries with >15% Total Body Surface Area (TBSA), standard sepsis criteria are not sufficiently specific. As a result, the current sepsis best practice alert utilized by our health system results in alarm fatigue and an inadequate escalation pathway for identifying sepsis in burn patients. A burn specific sepsis algorithm based off criteria recommended by the American Burn Association was developed and incorporated into the burn unit’s daily practice to promote early and actionable recognition of sepsis in burn patients with >15% TBSA.

**Methods:**

A quality improvement initiative was conducted from July 1, 2025, to August 30, 2025, involving seven patients with total body surface area (TBSA) burns greater than 15%. Interventions included the implementation of a burn specific algorithm and a questionnaire designed to guide staff in the early recognition and response to suspected burn sepsis. Results were gathered via questionnaire responses and patient chart audits. Unit wide education sessions were conducted to familiarize staff with the new algorithm and support consistent application of the protocol.

**Results:**

The health system's best practice alert fired on three occurrences, one of which warranted escalation. For that occurrence, burn sepsis criteria was met but no diagnosis was made. There were four occurrences where blood cultures were drawn but were not triggered by the health system's sepsis best practice alert. Fifty percent of those had growth and met burn specific sepsis criteria.

**Conclusions:**

The burn sepsis criteria demonstrated moderate sensitivity and good specificity, effectively identifying most cases but potentially missing early or atypical presentations. Limitations included low patient volume and a short implementation period, which restricted staff familiarity with the algorithm. Ongoing efforts include maintaining the questionnaire for continued access and education, continuing patient chart audits, integrating the "Burn Specific Sepsis Algorithm" into the health system's sepsis best practice alert, and developing a formal burn specific sepsis protocol.

**Applicability of Research to Practice:**

Standard sepsis criteria lack specificity in burn patients with >15% TBSA due to their hypermetabolic response, leading to alarm fatigue and missed or delayed escalation using the current health system's best practice alert. To address this gap, a burn specific sepsis algorithm based on American Burn Association guidelines was implemented in the burn unit to support timely recognition and intervention. While our data is limited results are promising that burn specific sepsis algorithm will support reduced alarm fatigue and effective identification of sepsis in burn patients.

**Funding for the study:**

N/A.

